# Rna analysis of the regulation of expression and alternative splicing in polycystic ovarian syndrome

**DOI:** 10.1080/15476286.2025.2606662

**Published:** 2025-12-24

**Authors:** Qi Zhang, Shujuan Zhu, Bin Jiang

**Affiliations:** a Department of Gynecology, The Third Xiangya Hospital, Central South University, Changsha, China; b Branch of National Clinical Research Center for Obstetrics and Gynecology, The Third Xiangya Hospital of Central South University, Changsha, China; c Center of Gynecological Disease and Reproductive Health, Furong Laboratory, Changsha, China

**Keywords:** Polycystic ovary syndrome, alternative splicing, nuclear factor-kappa B, bioinformatics analysis, regulatory networks

## Abstract

Polycystic ovary syndrome (PCOS) is a complex endocrine disorder whose pathophysiological mechanisms remain incompletely understood. Alternative splicing of transcription factors (TFs) may lead to significant functional consequences in the pathogenesis of PCOS. This study investigated genome-wide AS patterns and the expression of key TFs in PCOS to identify functionally relevant splicing events in a human dataset and validate them in a mouse model. Bioinformatics analysis of a PCOS RNA-seq dataset revealed 42 differentially spliced TFs, with enrichment in transcriptional regulation and metabolic pathways. Subsequent validation in a PCOS mouse model highlighted significant upregulation of *Nfkb1* and *Nfkb2*, along with a specific exon-skipping event in *Nfkb1* ;(Nfkb1-ES1496). Our findings demonstrate altered AS of critical TFs in PCOS, implicating dysregulated NF-κB signalling through splicing modulation as a potential contributor to the disorder, which may offer novel biomarker or therapeutic avenues.

## Introduction

Polycystic ovarian syndrome (PCOS) is a common endocrine disorder impacting women from adolescence through postmenopause [1–4]. Globally, the prevalence of PCOS is reported to be between 5% and 13% [1,2,5]. Women with PCOS face a heightened risk of infertility, endometrial cancer, obesity, insulin resistance, type 2 diabetes, impaired glucose tolerance, and various endocrine disorders [6–8]. Patients with PCOS often present with reproductive, endocrine, metabolic, cardiovascular, and dermatological symptoms, commonly marked by androgen excess, ovulatory dysfunction, and polycystic ovarian morphology [7,9]. The latest PCOS management guidelines indicate that only symptomatic treatment is available for patients, as no specific therapy exists [5,8].

The precise cause of PCOS remains unclear, likely involving a complex interaction of genetic, metabolic, and environmental factors [5,10]. PCOS exhibits familial clustering and high heritability, and approximately twenty genes associated with susceptibility to PCOS have been identified through genome-wide association studies [11,12]. Environmental factors can alter epigenetic mechanisms, such as DNA methylation, histone modification, and non-coding RNAs, thereby amplifying the effects of risk genotypes and heightening disease susceptibility [11–13]. Despite numerous studies on PCOS, the specific pathogenic mechanisms remain unclear.

Alternative splicing (AS) of pre-mRNA is essential for gene regulation, enhancing transcriptome and proteome diversity, and is crucial for tissue development, differentiation, and key cellular pathways in higher eukaryotes [14,15]. AS can produce mRNA with diverse untranslated regions or coding sequences via mechanisms such as constitutive splicing, exon skipping (ES) or cassette exon (CE), intron retention (IR), mutually exclusive exons (MXE), selection of alternative 3′ or 5′ splice sites (A3SS/A5SS), and alternative first or last exons (AFE/ALE) [15,16]. All of these mechanisms can influence mRNA stability, localization, and translation [15].

Transcription factors (TFs) are key regulators of gene expression, and their alternative splicing may lead to significant functional consequences in complex diseases such as PCOS [17–19]. This study utilized RNA sequencing data from the Gene Expression Omnibus database to analyse differences in AS events between individuals with PCOS and healthy controls using bioinformatics techniques. We investigated AS patterns and transcription factor expression levels to offer new insights into PCOS pathophysiology.

## Materials and methods

### Dataset acquisition and reads alignment

The RNA-seq dataset GSE168404 was sourced from the Gene Expression Omnibus Database at the National Center for Biotechnology Information. This dataset, generated using the GPL16791 platform (Illumina HiSeq 2500), includes GC samples from five patients diagnosed with PCOS and five corresponding control subjects [20]. The clean reads were mapped to the human GRCh38 genome using HISAT2 [21]. Uniquely mapped reads were used to calculate read counts and fragments per kilobase of exon per million mapped reads (FPKM) for each gene.

### Differentially expressed gene (DEG) analysis　

Gene expression levels were measured using FPKM values. Genes showing differential expression were detected using the DESeq2 software, applying cut-off thresholds of |log2 fold change| ≥1 and p-value < 0.05.

### Alternative splicing (AS) analysis by splice sites usage variation analysis (SUVA)

The Splice Sites Usage Variation Analysis (SUVA) pipeline was employed to define and quantify alternative splicing (AS) events [22]. Differential splicing between each pair of diseased and control tissues was analysed. The frequency and read proportion of SUVA AS events (pSAR) were determined for each AS event. SUVA defines five distinct types of AS events [22]: alt3p and alt5p, in which one splice junction (SJ) is common while the other is alternative; olp and contain, where both SJs differ when the alternatively SJ is either overlapping or contained; and ir, where both SJs are either utilized or skipped. In SUVA, a SJ with only one AS event is defined as a simple event, while a SJ with more than two AS events is defined as a complex event. The splicing ratio was calculated as SJ reads _short_/(SJ reads _short_ + SJ reads _long_) [22].

### Functional enrichment analysis

Functional categories related to the AS were identified using Gene Ontology (GO) and the Kyoto Encyclopedia of Genes and Genomes (KEGG) pathway analyses via the KOBAS 2.0 server. The findings were considered significantly enriched when the p-value was less than 0.05.

### Animal experiment

The procedures for establishing a comprehensive PCOS mouse model were outlined in our earlier publication [23]. Seven-week-old female C57BL/6J mice were obtained from SJA Laboratory Animal Co. Ltd (Hunan, China) and acclimated for one week under a 12-hour light/dark cycle at 24 ± 3°C and 45 ± 2% humidity. Vaginal smears were performed daily at 8:00 a.m. throughout the feeding period to monitor the oestrous cycle. A total of ten mice exhibiting a regular oestrous cycle were randomly assigned to two groups: the PCOS group and control group. Mice in the PCOS group were administered daily subcutaneous injections of 6 mg dehydroepiandrosterone (DHEA) per 100 g body weight, supplemented with 0.2 ml injectable soybean oil. Conversely, the control group mice were administered 0.2 ml of injectable soybean oil on a daily basis. Then mice displaying continuous keratosis of vaginal epithelial cells within the PCOS group were euthanized with cervical dislocation, along with those from the control group. Ovaries from two randomly chosen mice per group underwent embedding, sectioning, and haematoxylin and eosin (H&E) staining to evaluate histological changes and confirm PCOS induction. The H&E staining results are provided in Supplementary Figure S1. The ovaries were stored at −80°C for Western blotting analysis, RNA extraction, sequencing, and reverse transcription quantitative polymerase chain reaction (RT-qPCR).

### Western blotting

Protein concentration in ovarian tissues was measured using the Bradford assay after protein extraction (Beyotime, China). Protein samples were separated using sodium dodecyl sulphate polyacrylamide gel electrophoresis (SDS-PAGE). The proteins that had been separated were then transferred onto polyvinylidene fluoride (PVDF) membranes. These membranes were incubated with specific antibodies to identify the target proteins. The antibodies utilized in this study were as follows: NFKB1 (1:1000, ABclonal), NFKB2 (1:1000, ABclonal), and glyceraldehyde-3-phosphate dehydrogenase (GAPDH, dilution ratio 1꞉1 000, Proteintech).

### RNA extraction, sequencing and analysis

Ovarian tissues were meticulously dissected into small fragments and subsequently homogenized in TRIzol reagent. Total RNA was quantified using a NanoDrop ND-1000 spectrophotometer (Thermo Fisher Scientific, USA). RNA-seq library preparation was performed using 1–2 μg of total RNA per sample. Total RNA was isolated and fragmented utilizing the Poly(A) mRNA Magnetic Isolation Module (New England Biolabs, Ipswich, MA, USA) and the KAPA Stranded RNA-Seq Kits (Roche, Basel, Switzerland). The RNA was converted to single-stranded cDNA, then amplified, purified, and quantified for sequencing on the Illumina NovaSeq 6000 (San Diego, California, USA).

### Reverse transcription quantitative polymerase chain reaction (RT-qPCR)

Supplementary Table S1 contains details about the RT-qPCR primers. The RNA underwent reverse transcription to cDNA employing the SuperScript III Reverse Transcriptase kit (Invitrogen, Waltham, MA, USA). Quantitative polymerase chain reaction (qPCR) was performed using the Bioer 9600 FQD-96A system and the Universal SYBR Green Fast qPCR Mix kit (Transgenbiotech, China). The cycling protocol began with a pre-denaturation at 95°C for 10 minutes, followed by 40 cycles consisting of a 10-second denaturation at 95°C and a 1-minute annealing and extension at 60°C. Cycle threshold (Ct) values were determined using the 2^−△△CT^ method, normalizing target gene RNA expression levels to GAPDH.

### Statistical analysis

Principal component analysis (PCA) was performed with the R package factoextra (https://cloud.r-project.org/package=factoextra) to demonstrate sample clustering. Clustering based on Euclidean distance was conducted using the heatmap package in R (https://cran.r-project.org/web/packages/pheatmap/index.html). The Student’s t-test was utilized for the comparative analysis between the two groups. RT-qPCR assay results, expressed as mean ± standard deviation (SD), were analysed for group differences using the Student’s t-test or Mann – Whitney U test with SPSS version 21.0 (IBM Corp, Armonk, NY, USA). A p-value below 0.05 was considered statistically significant.

## Results

### Transcriptome analysis of alternative splicing between PCOS and healthy samples

**Figure 1.**
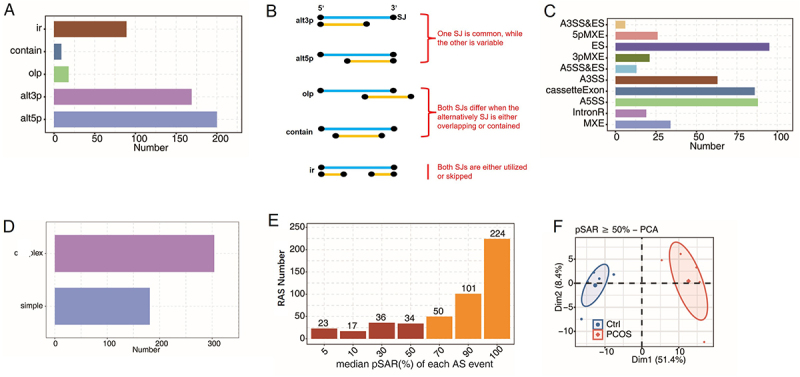
Transcriptome analysis of as between PCOS and healthy samples.

**Figure 2.**
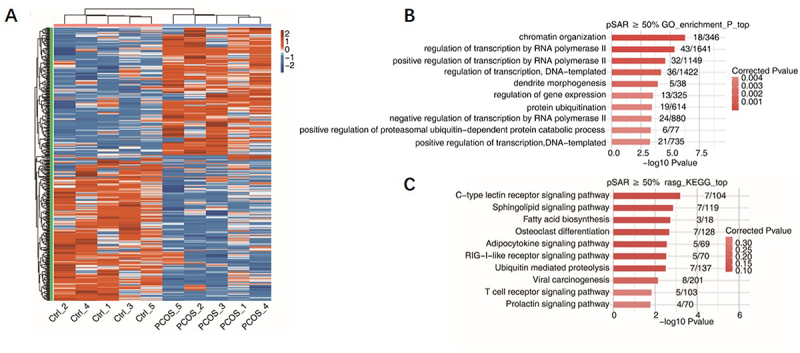
Functional analysis of as between PCOS and control samples.

To investigate the differences in AS events between individuals with PCOS and healthy controls, we utilized SUVA software to analyse and identify the AS events in the two groups. As illustrated in Figures 1(A–C), the primary types of AS events identified were ES, A5SS, CE, and A3SS. Complex AS events are more prevalent, identified in all AS events in Figure 1(D). To differentiate between major and minor transcripts, the pSAR for each AS event was estimated. Our analysis revealed that more than half of the AS events originated from major transcripts (pSAR ≥50%), as shown in Figure 1(E). All subsequent analyses in this research were performed utilizing AS events characterized by a pSAR of 50%. Principal component analysis (PCA) was employed using the AS for pSAR ≥50% in Figure 1(F), and the results illustrates that applying PCA to the filtered AS events successfully differentiated the PCOS group from the control group. Comparable findings were noted in the heatmap produced from the hierarchical cluster analysis presented in Figure 2(A). We conducted a further analysis of the potential functional implications of the differentially expressed AS events by exploring the GO and KEGG databases, focusing on the associated biological pathways. The data presented in Figure 2(B) illustrate the top ten pathways identified in GO enrichment analysis related to transcriptional regulation. These pathways encompass chromatin organization and the regulation of transcription by both RNA polymerase and DNA templating. The data presented in Figure 2(C) show the top ten pathways enriched in the KEGG database, primarily including immune and metabolic pathways. Transcriptional regulation and metabolic pathways were identified as key pathways involved in the AS events of patients with PCOS.

(A) A bar plot illustrating the number of AS events detected by SUVA in each group. In the figure, alt3p and alt5p, in which one splice junction (SJ) is common while the other is alternative; olp and contain, where both SJs differ when the alternatively SJ is either overlapping or contained; and ir, where both SJs are either utilized or skipped.

(B) The schematic diagrams for each type of splicing event are shown in (A).

(C) SJs constituting AS events detected by SUVA were annotated to classical AS event types. The number of each classical AS event type was displayed using a bar plot. ES: exon skipping; A3SS/A5SS: alternative 5’or 3’ splice site; 3pMXE/5pMXE: mutually exclusive 5’UTRs or 3’UTRs; IntronR: intron retention; MXE: mutually exclusive exons.

(D) A bar plot showing AS alongside complex and simple AS.

(E) A bar plot illustrating AS with varying the pSAR values. AS with a pSAR ≥50% was labelled.

(F) Principal component analysis (PCA) was conducted using the AS for pSAR ≥50%. The ellipse representing each group indicates the confidence interval.

(A) The heatmap illustrating the splicing ratio of AS (pSAR ≥50%).

(B) A bar plot showing the most enriched GO biological process results for the AS (pSAR ≥50%).

(C) A bar plot presenting the most enriched KEGG pathway results for the AS (pSAR ≥50%).

### Transcription factors with alternative splicing coordinately regulate gene expression in PCOS samples

**Figure 3.**
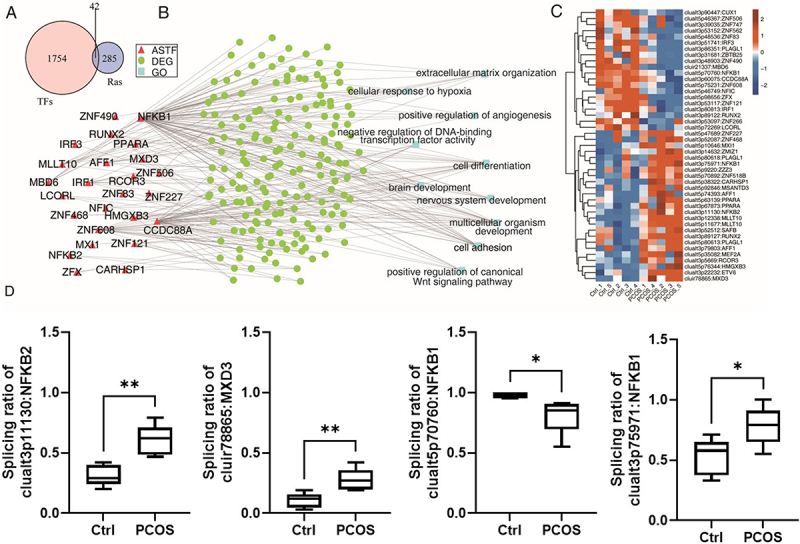
Differential TFs coordinate the regulation of as events in the PCOS and control groups.

TFs, crucial for gene expression regulation, were identified concerning alternative splicing TFs (AS-TFs) and differentially expressed genes (DEGs). We identified a total of 42 AS-TFs in this study (Figure 3(A)). Figure 3(B) displays the functional enrichment and co-expression analysis of AS-TFs. The AS-TFs-DEGs regulatory networks primarily target extracellular matrix organization, cellular hypoxia responses, angiogenesis enhancement, transcription factor activity regulation, cell differentiation and adhesion, brain and nervous system development, multicellular organism development, and the activation of the canonical Wnt signalling pathway. The heatmap generated from the hierarchical cluster analysis is presented in Figure 3(C). Furthermore, we presented the splicing ratio and statistical differences of AS-TFs for some important genes in Figure 3(D). In addition, we presented visualized read distribution plots for clualt3p11130: NFKB2, clualt5p70760: NFKB1, and clualt3p75971: NFKB1 in Supplementary Figure S2, which are among the most representative AS-TFs.

(A) Venn diagram showing the overlap of AS-TFs among TFs and AS events.

(B) The network plot illustrating the differentially expressed genes (DEGs) regulated by AS-TFs.

(C) Hierarchical clustering heatmap of AS-TFs expression levels.

(D) Bar plots showing the splicing ratio and statistical differences of AS-TFs for some important genes from (A)

### Functional analysis of Nfkb in PCOS

**Figure 4.**
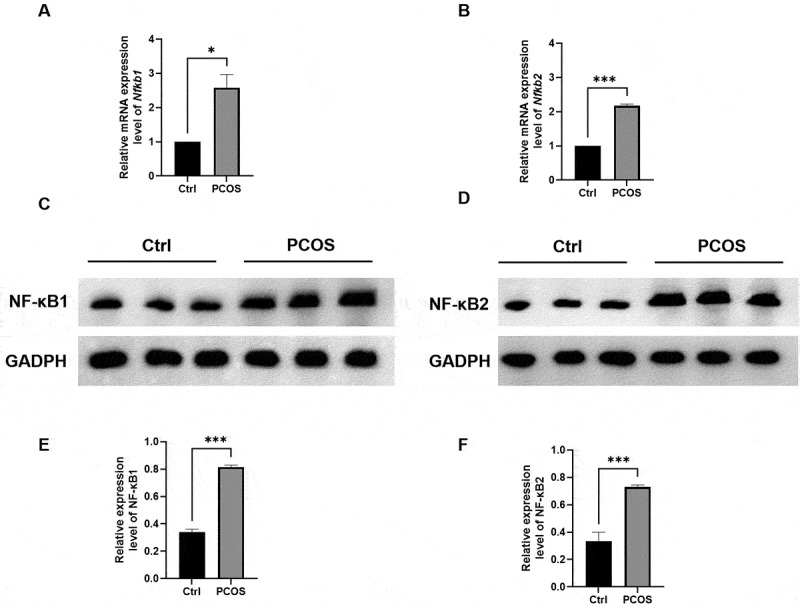
Functional analysis of *Nfkb1* and *Nfkb2* detected by RT-qPCR and Western blotting.

As one of the top immune-related pathways identified in the functional enrichment analyses and combined with the analysis of alternative splicing (AS) events, the nuclear factor-kappa B (NF-κB) pathway was further studied. We investigated the mRNA and protein levels of *Nfkb1* and *Nfkb2* using RT-qPCR (Figure 4(A,B)) and Western blotting (Figure 4(C–F)) in mice with a successfully established PCOS model (Supplementary Figure S1). The expression levels of *Nfkb1*and *Nfkb2* were significantly elevated in PCOS mice compared to the control group (*p* < 0.05 and *p* < 0.001, respectively). However, there appears to be no significant difference in the gene expression levels of *Nfkb1* and *Nfkb2* in humans sequencing data.

(A) and (B): The comparison of *Nfkb1* and *Nfkb2* mRNA expression levels between PCOS and control mice.

(C) and (D): Representative image of NF-κB protein expression levels in the ovary detected in PCOS and control mice.

(E) and (F): The comparison of NF-κB protein expression levels between PCOS and control mice.

*P<0.05, ***P<0.001

**Figure 5.**
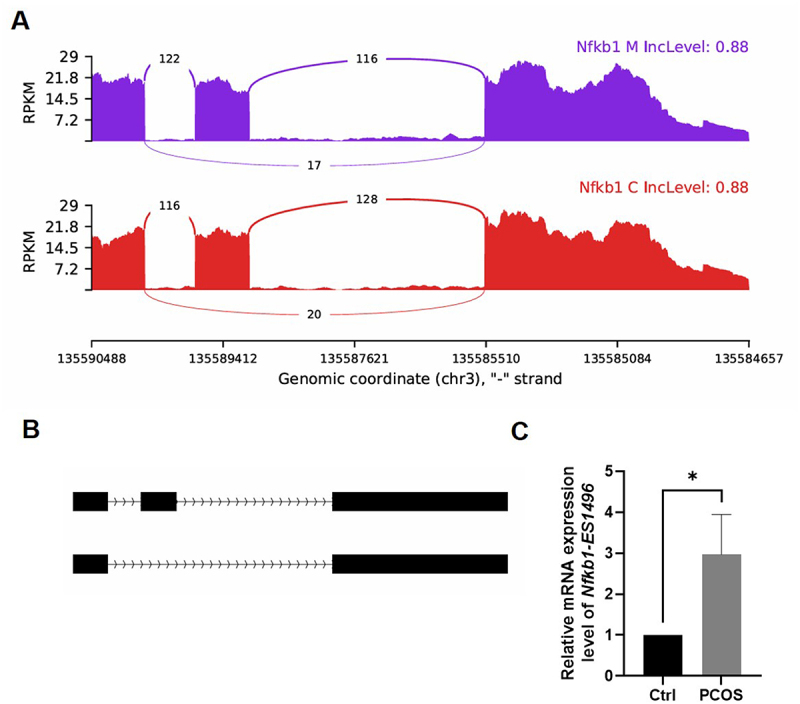
Functional analysis of the as event of *Nfkb1-ES1496.*

**Figure 6.**
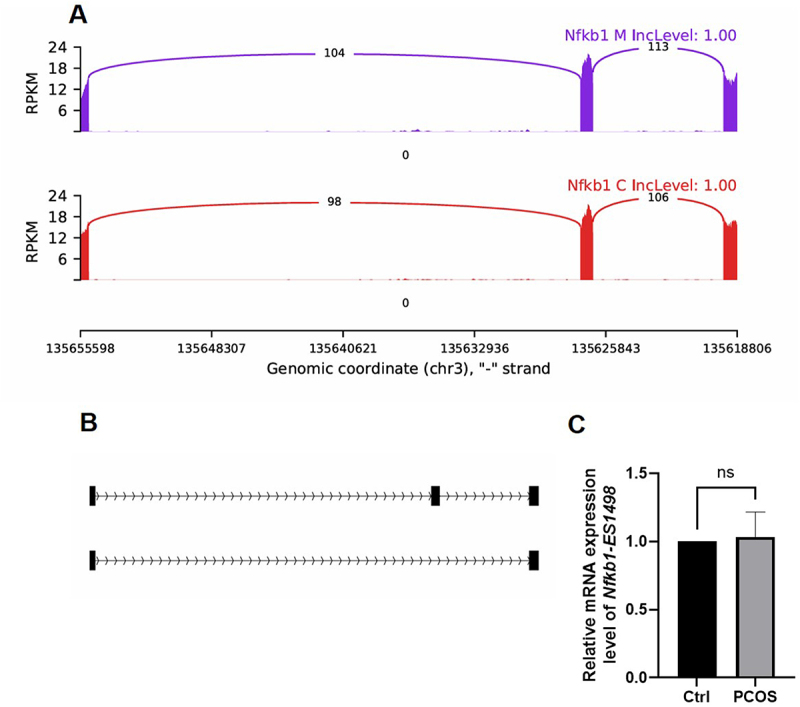
Functional analysis of the as event of *Nfkb1-ES1498.*

We then combined RNA sequencing and AS events analysis (Supplementary Figure S3C) to validate whether AS events of *Nfkb1* and *Nfkb2* are involved in the development of PCOS. It appears that there are no AS events related to *Nfkb2* transcription in mice. However, the two AS exon skipping events of *Nfkb1*, designated as *Nfkb1-ES1496* (Figure 5(A,B)) and *Nfkb1-ES1498* (Figure 6(A,B)), were further verified by RT-qPCR. Notably, the expression of *Nfkb1-ES1498* did not differ in AS events between the PCOS group and the control group mice (Figure 6(C)). The PCOS group exhibited significantly higher *Nfkb1-ES1496* expression compared to the control group (*p* < 0.05, Figure 5(C)).

(A) The read distribution of the AS event of *Nfkb1-ES1496* is plotted with PCOS (upper panel in purple) and the control sample (lower panel in red).

(B) The schematic diagrams illustrate the structures of the AS event.

(C) The RT-qPCR validation of *Nfkb1-ES1496* is presented.

(A) The read distribution of the AS event associated with *Nfkb1-ES1498* is illustrated, with the data for polycystic ovary syndrome (PCOS) represented in the upper panel in purple, and the control sample depicted in the lower panel in red.

(B) The schematic diagrams show the structures of the AS event.

(C) The validation of *Nfkb1-ES1498* using RT-qPCR is documented.

**Figure 7.**
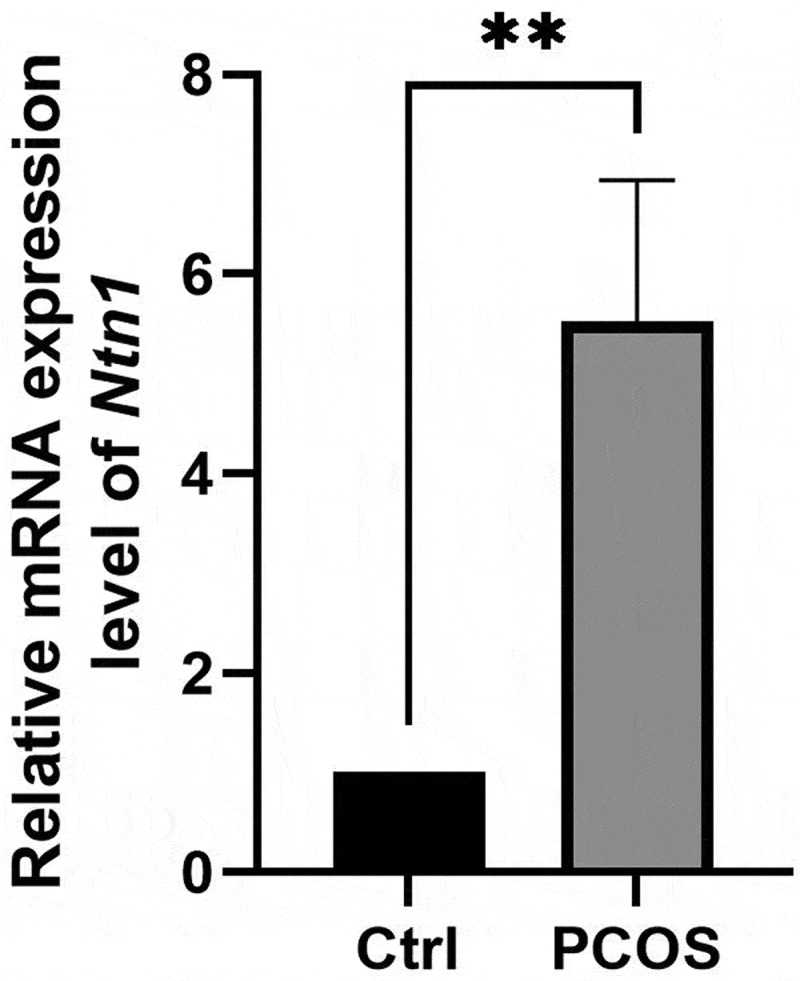
The comparison of *Ntn1* mRNA expression levels between PCOS and control mice.

Our RNA sequencing data identified a total of 217 DEGs between the control and PCOS mice, with 155 genes upregulated and 62 downregulated (Supplementary Figure S3A and S3B). By combining the genes that may be targets of *Nfkb1*, as identified in the Transcriptional Regulatory Relationships Unraveled by Sentence-based Text Mining (TRRUST) database, we identified five genes that may be upregulated by *Nfkb1* (Table 1). We then identified the expression levels of Netrin-1 (*Ntn1)* were significantly elevated in PCOS mice compared to the control group in (Figure 7) (*p* < 0.01).

**Table 1.** Significantly differentially expressed gene regulated by *Nfkb1.*

Gene symbol	Regulation	Log2Fold change	Fold change	p value
*Igf2bp2*	up	0.618895187	1.535698696	0.0221576
*Ltc4s*	up	0.764110672	1.698322769	0.0161822
*Slc2a1*	up	0.89842006	1.864023515	0.0389113
*Ccnd3*	up	0.953798125	1.936965322	0.0280602
*Ntn1*	up	1.165690898	2.243406252	0.0412993

** P<0.01.

## Discussion

PCOS recognized as a common endocrine disorder, which may exert considerable adverse effects on women’s health. The pathogenic mechanisms associated with PCOS remain inadequately understood. Recent studies suggest that alternative splicing events of transcription factors, which control gene expression at the transcriptional level, may play a significant role in the development and progression of PCOS [17–19].

AS events have been demonstrated to be integral to tissue development and associated pathologies, such as germ cell maturation and female infertility [15,24]. AS describes the process by which one gene can produce multiple transcript variants, leading to greater diversity in the proteome [15,16]. In a physiological state, numerous AS events occur, and the transition between AS isoforms contributes to the acquisition of specific functions and characteristics of tissues. A single AS event undergoes coordinated changes during development, forming an alternative splicing network. AS enables a single gene to generate diverse mRNA isoforms, leading to proteins with different structures and functions. Nevertheless, some AS events yield non-coding transcripts that are not translated into functional proteins [14,15]. RNA stability might be compromised, and alterations in mRNA localization could impede the proper functioning of transcripts or proteins [14,15].

AS events are essential in tissue development and associated diseases, such as germ cell development and female infertility [15,24]. Research indicates that alternative splicing events in certain candidate genes are linked to women with PCOS [17–19]. However, studies examining the relationship between PCOS and AS are currently rare and warrant further investigation. We analysed RNA sequencing data from individuals diagnosed with PCOS and healthy control subjects to examine the AS patterns and expression levels of critical transcription factors using the GSE168404 dataset in this study. Transcriptional regulation and metabolic pathways were identified as key pathways involved in the AS events of patients with PCOS through GO and KEGG enrichment analyses, consistent with previous research [13,25].

We then identified a total of 42 AS-TFs that predominantly pertain to developmental processes, cellular responses to hypoxia, and cell differentiation, as established through functional enrichment and co-expression analyses. These findings suggest that AS of TFs may contribute to PCOS through diverse biological processes beyond the NF-κB pathway. The potential candidate AS-TFs identified herein, which may serve as critical regulatory functions in PCOS, were subjected to further investigation and analysis.

Nuclear factor-kappa B (NF-κB) is a transcription factor that regulates diverse biological processes, including inflammation, differentiation, proliferation, apoptosis, and cell survival. It simultaneously mediates cellular responses to stress, ischaemia, and adaptive mechanisms across diverse cell types and organs, playing a crucial role in various physiological and pathophysiological processes [26,27]. The NF-κB transcription factor complex in mammals consists of five members: RelA (p65), RelB, c-Rel, NF-κB1 (p50), and NF-κB2 (p52). In resting cells, the members are inactivated by binding with inhibitory proteins (IκB), and different combinations of these members are responsible for the regulation of various target genes. Upon receiving certain signals, NF-κB is activated and moves to the nucleus to control gene expression [28,29].

The role of chronic inflammation in the development of PCOS has been emphasized in recent studies [27,30,31]. Patients with PCOS are typically characterized by increased levels of NF-κB expression and decreased expression of IκB in their serum and ovarian tissues [27,30,32]. NF-κB pathway has also been verified to be associated with cardiac inflammation and kidney injury related to polycystic ovary syndrome (PCOS) [26,33]. In the human RNA-seq data, *Nfkb1* and *Nfkb2* also showed upregulated trends, though not statistically significant, possibly due to sample size limitations. The comprehension of the AS of *Nfkb* remains constrained, especially regarding its implications in PCOS. Our study found significantly increased expression levels of *Nfkb1* and *Nfkb2* in the PCOS mouse model compared to the control group (*p* < 0.05 and *p* < 0.001), supporting previous research findings [27,31]. We then verified that *Nfkb1-ES1496* expression, an exon skipping event linked to AS, was significantly higher in the PCOS group than in the control group. It appears that AS events can occur independently of changes in overall gene expression.

*Ntn*1 was initially identified for its role in axon guidance during nervous system development, which may also promote the retention of adipose tissue macrophages and contribute to insulin resistance in obesity [34,35]. We found that the expression levels of *Ntn*1, a potential target gene of *Nfkb1*, were significantly higher in PCOS mice compared to the control group (*p* < 0.01). Since no related studies have been published previously, this topic still needs further investigation.

This study employed an integrated bioinformatics and experimental approach to investigate AS’s role in the NF-κB signalling pathway concerning PCOS pathogenesis, aiming to identify new diagnostic and therapeutic biomarkers. However, we acknowledge several limitations of this study. The sample size was relatively small, which may limit the generalizability of the findings. Additionally, the mouse model may not fully replicate human PCOS due to species and tissue differences. Furthermore, protein-level validation and functional assays were not well performed, which should be addressed in future research.

In conclusion, our research revealed the differences in the alternative splicing of critical transcription factors between subjects with PCOS and control subjects. The AS in the NF-κB signalling pathway could play a crucial role in PCOS pathophysiology, indicating its potential as a new biomarker or therapeutic target.

## Supplementary Material

Supplemental Material

## Data Availability

The data and material presented in this manuscript is available from the corresponding author on reasonable request. The raw sequence data reported in this paper have been deposited in the Genome Sequence Archive (Genomics, Proteomics & Bioinformatics 2025) [36] in National Genomics Data Center (Nucleic Acids Res 2025), China National Center for Bioinformation/Beijing Institute of Genomics, Chinese Academy of Sciences [37] (GSA: CRA033411) that are publicly accessible at https://ngdc.cncb.ac.cn/gsa.

## References

[1] Stener-Victorin E. Polycystic ovary syndrome. Nat Rev Dis Primers. 2024;10(1):27. 38637590 10.1038/s41572-024-00511-3

[2] Yun C, Yan S, Liao B, et al. The microbial metabolite agmatine acts as an FXR agonist to promote polycystic ovary syndrome in female mice. Nat Metab. 2024;6(5):947–962. doi: 10.1038/s42255-024-01041-8 38769396

[3] Bahri Khomami M, Hashemi S, Shorakae S, et al. Systematic review and meta-analysis of birth outcomes in women with polycystic ovary syndrome. Nat Commun. 2024;15(1):5592. doi: 10.1038/s41467-024-49752-6 38965241 PMC11224419

[4] Bahri Khomami M, Shorakae S, Hashemi S, et al. Systematic review and meta-analysis of pregnancy outcomes in women with polycystic ovary syndrome. Nat Commun. 2024;15(1):5591. doi: 10.1038/s41467-024-49749-1 38965226 PMC11224312

[5] Teede HJ, Tay CT, Laven J, et al. Recommendations from the 2023 international evidence-based guideline for the assessment and management of polycystic ovary syndrome. Fertil Steril. 2023;120(4):767–793. doi: 10.1016/j.fertnstert.2023.07.025 37589624

[6] Samarasinghe SNS. Impact of insulin sensitization on metabolic and fertility outcomes in women with polycystic ovary syndrome and overweight or obesity-a systematic review, meta-analysis, and meta-regression. Obes Rev. 2024;25(7):e13744. 38572616 10.1111/obr.13744

[7] van der Ham K. Anti-Mullerian hormone as a diagnostic biomarker for polycystic ovary syndrome (PCOS) and polycystic ovarian morphology (PCOM): a systematic review and meta-analysis. Fertil Steril. 2024;122(4): 727–739. 38944177 10.1016/j.fertnstert.2024.05.163

[8] Dason ES. Diagnosis and management of polycystic ovarian syndrome. CMAJ. 2024;196(3):E85–E94. 38286488 10.1503/cmaj.231251 PMC10833093

[9] Pinto J, Cera N, Pignatelli D. Psychological symptoms and brain activity alterations in women with PCOS and their relation to the reduced quality of life: a narrative review. J Endocrinol Invest. 2024;47(7):1–22. 10.1007/s40618-024-02329-y PMC11196322 38485896

[10] Stanczak NA, Grywalska E, Dudzinska E. The latest reports and treatment methods on polycystic ovary syndrome. Ann Med. 2024;56(1):2357737. 38965663 10.1080/07853890.2024.2357737 PMC11229724

[11] Joham AE, Norman RJ, Stener-Victorin E, et al. Polycystic ovary syndrome. Lancet Diabetes Endocrinol. 2022;10(9):668–680. doi: 10.1016/S2213-8587(22)00163-2 35934017

[12] Chen Y, Wang G, Chen J, et al. Genetic and epigenetic landscape for drug development in polycystic ovary syndrome. Endocr Rev. 2024;45(4):437–459. doi: 10.1210/endrev/bnae002 38298137

[13] Mimouni NEH, Paiva I, Barbotin A-L, et al. Polycystic ovary syndrome is transmitted via a transgenerational epigenetic process. Cell Metab. 2021;33(3):513–530 e8. doi: 10.1016/j.cmet.2021.01.004 33539777 PMC7928942

[14] Marasco LE, Kornblihtt AR. The physiology of alternative splicing. Nat Rev Mol Cell Biol. 2023;24(4):242–254. doi: 10.1038/s41580-022-00545-z 36229538

[15] Tao Y, Zhang Q, Wang H, et al. Alternative splicing and related RNA binding proteins in human health and disease. Signal Transduct Target Ther. 2024;9(1):26. doi: 10.1038/s41392-024-01734-2 38302461 PMC10835012

[16] Wan R, Bai R, Zhan X, et al. How is precursor messenger RNA spliced by the spliceosome? Annu Rev Biochem. 2020;89:333–358. 31815536 10.1146/annurev-biochem-013118-111024

[17] Luo J, Ye H, Hao L, et al. SRSFs mediate the function of AR in the ovarian granulosa cells of patients with PCOS. Genes Dis. 2021;8(1):94–109. doi: 10.1016/j.gendis.2019.09.005 33569518 PMC7859457

[18] Zhao F, Wu L, Wang Q, et al. Insulin-like growth factor 2 mRNA-binding protein 2-regulated alternative splicing of nuclear factor 1 C-type causes excessive granulosa cell proliferation in polycystic ovary syndrome. Cell Prolif. 2022;55(4):e13216. doi: 10.1111/cpr.13216 35293050 PMC9055906

[19] Wang F, Pan J, Liu Y, et al. Alternative splicing of the androgen receptor in polycystic ovary syndrome. Proc Natl Acad Sci U S A. 2015;112(15):4743–4748. doi: 10.1073/pnas.1418216112 25825716 PMC4403157

[20] Zhao R, Jiang Y, Zhao S, et al. Multiomics analysis reveals molecular abnormalities in granulosa cells of women with polycystic ovary syndrome. Front Genet. 2021;12:648701. 34084179 10.3389/fgene.2021.648701 PMC8168535

[21] Kim D, Langmead B, Salzberg SL. Hisat: a fast spliced aligner with low memory requirements. Nat Methods. 2015;12(4):357–360. doi: 10.1038/nmeth.3317 25751142 PMC4655817

[22] Cheng C, Liu L, Bao Y, et al. Suva: splicing site usage variation analysis from RNA-seq data reveals highly conserved complex splicing biomarkers in liver cancer. RNA Biol. 2021;18(sup1):1–15. doi: 10.1080/15476286.2021.1940037 PMC8682974 34152934

[23] Zou L, Li W, Xu D, et al. Alteration of the n(6)-methyladenosine methylation landscape in a mouse model of polycystic ovary syndrome. J Ovarian Res. 2023;16(1):157. doi: 10.1186/s13048-023-01246-7 37550765 PMC10408202

[24] Feng S, Li J, Wen H, et al. Hnrnph1 recruits Ptbp2 and Srsf3 to modulate alternative splicing in germ cells. Nat Commun. 2022;13(1):3588. doi: 10.1038/s41467-022-31364-7 35739118 PMC9226075

[25] Ducreux B, Ferreux L, Patrat C, et al. Overview of gene expression dynamics during human oogenesis/folliculogenesis. Int J Mol Sci. 2023;25(1):33. doi: 10.3390/ijms25010033 38203203 PMC10778858

[26] Olaniyi KS, Areloegbe SE. Suppression of PCSK9/NF-kB-dependent pathways by acetate ameliorates cardiac inflammation in a rat model of polycystic ovarian syndrome. Life Sci. 2022;300:120560. doi: 10.1016/j.lfs.2022.120560 35452635

[27] Kumariya S, Ubba V, Jha RK, et al. Autophagy in ovary and polycystic ovary syndrome: role, dispute and future perspective. Autophagy. 2021;17(10):2706–2733. doi: 10.1080/15548627.2021.1938914 34161185 PMC8526011

[28] Zhang T, Ding C, Chen H, et al. m(6)a mRNA modification maintains colonic epithelial cell homeostasis via NF-kappaB-mediated antiapoptotic pathway. Sci Adv. 2022;8(12):eabl5723. doi: 10.1126/sciadv.abl5723 35333576 PMC8956260

[29] Spinelli G, Biddeci G, Artale A, et al. A new p65 isoform that binds the glucocorticoid hormone and is expressed in inflammation liver diseases and COVID-19. Sci Rep. 2021;11(1):22913. 34824310 10.1038/s41598-021-02119-z PMC8617276

[30] Khodir SA, Sweed E, Motawea SM, et al. Diacerein and myo-inositol alleviate letrozole-induced PCOS via modulation of HMGB1, SIRT1, and NF-kB: a comparative study. Naunyn Schmiedebergs Arch Pharmacol. 2024;398(4):4179–4197. doi: 10.1007/s00210-024-03497-7 39432066 PMC11978706

[31] Zhang W, Wu F. Linoleic acid induces human ovarian granulosa cell inflammation and apoptosis through the ER-FOXO1-ROS-NFkappaB pathway. Sci Rep. 2024;14(1):6392. doi: 10.1038/s41598-024-56970-x 38493198 PMC10944505

[32] Yu S, Wang B, Qin Y, et al. Mesencephalic astrocyte-derived neurotrophic factor ameliorates inflammatory response in polycystic ovary syndrome via inhibiting TLR4-NF-kappaB-NLRP3 pathway. Biochem Biophys Res Commun. 2024;707:149782. 38493745 10.1016/j.bbrc.2024.149782

[33] Ye HY, Song YL, Ye WT, et al. Serum granulosa cell-derived TNF-alpha promotes inflammation and apoptosis of renal tubular cells and PCOS-related kidney injury through NF-kappaB signaling. Acta Pharmacol Sin. 2023;44(12):2432–2444. 37507430 10.1038/s41401-023-01128-0 PMC10692080

[34] Ramkhelawon B, Hennessy EJ, Ménager M, et al. Netrin-1 promotes adipose tissue macrophage retention and insulin resistance in obesity. Nat Med. 2014;20(4):377–384. 24584118 10.1038/nm.3467 PMC3981930

[35] Sharma M, Schlegel M, Brown EJ, et al. Netrin-1 alters adipose tissue macrophage fate and function in obesity. Immunometabolism. 2019;1(2). doi: 10.20900/immunometab20190010 PMC6699780 31428465

[36] Zhang 张思思 S. The GSA family in 2025: a broadened sharing platform for multi-omics and multimodal data. Genomics Proteomics Bioinf. 2025;23(4):qzaf072. 10.1093/gpbjnl/qzaf072 PMC12451262 40857552

[37] Members C-N. Database resources of the National Genomics Data Center, China National Center for Bioinformation in 2025. Nucleic Acids Res. 2025;53(D1):D30–D44. 39530327 10.1093/nar/gkae978 PMC11701749

