# Supplementary figures and images for: Rna analysis of the regulation of expression and alternative splicing in polycystic ovarian syndrome
Source: RNA Biol. 2025 Dec 24;23(1):1–12. doi: 10.1080/15476286.2025.2606662 (PMC12758344; doi:10.1080/15476286.2025.2606662)

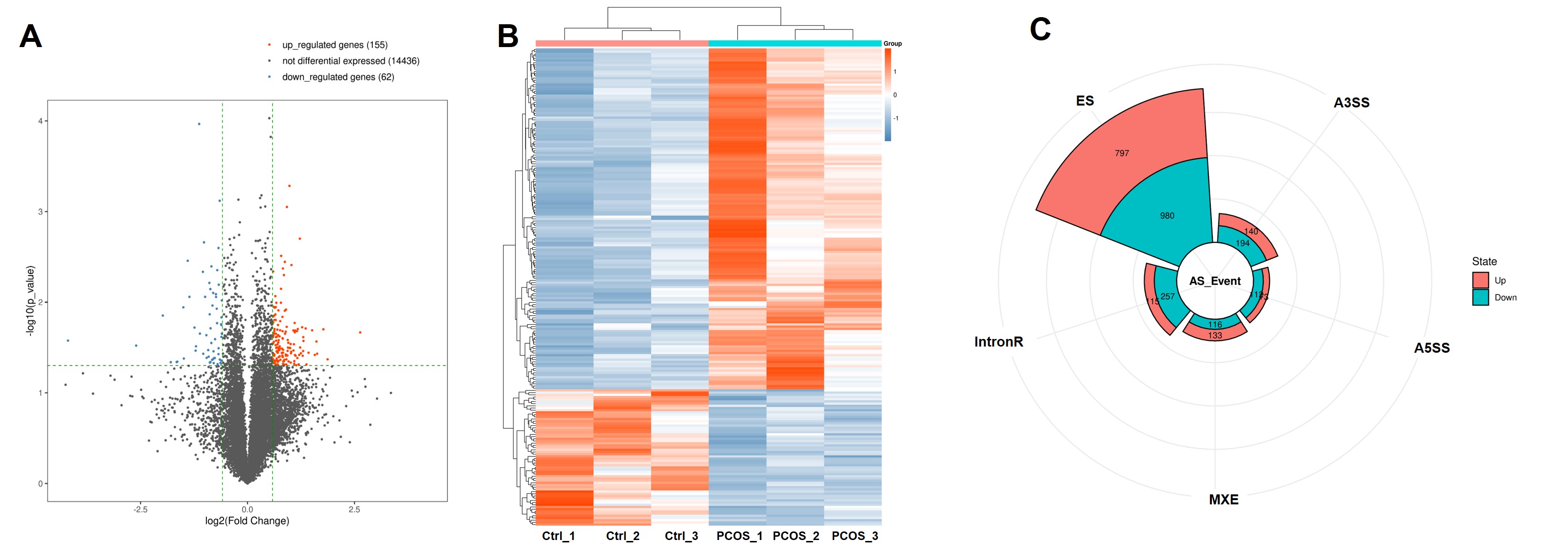

Supplement: Supplemental Material [file KRNB_A_2606662_SM3625.zip › Supplementary figS3.jpg]

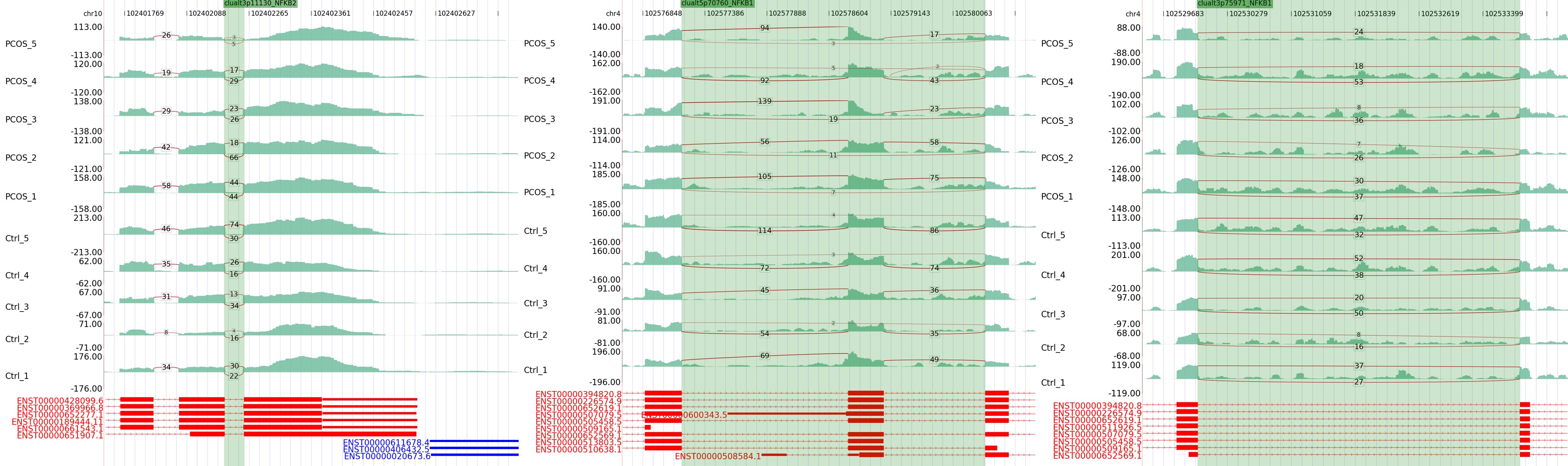

Supplement: Supplemental Material [file KRNB_A_2606662_SM3625.zip › Supplementary figS2.jpg]

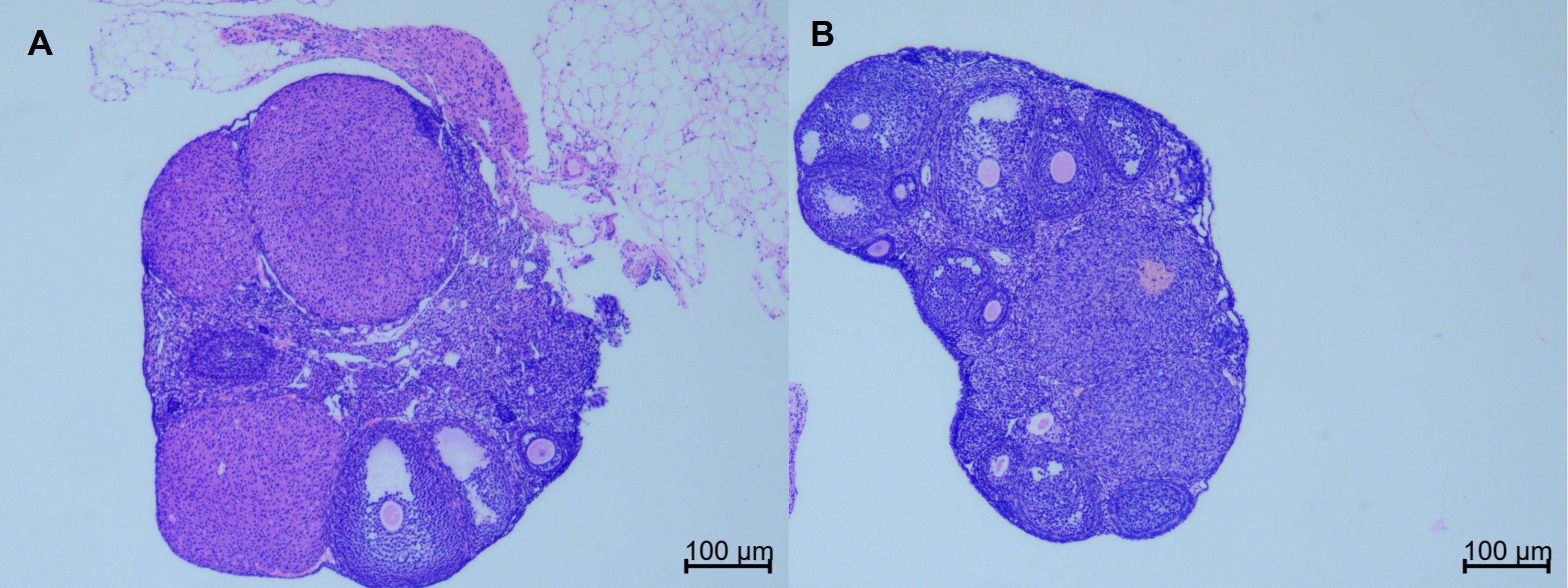

Supplement: Supplemental Material [file KRNB_A_2606662_SM3625.zip › Supplementary figS1.jpg]
